# Falsch- und Desinformation in sozialen Medien: Ansätze zur Minimierung von Risiken in digitaler Kommunikation über Gesundheit

**DOI:** 10.1007/s00103-024-03836-2

**Published:** 2024-02-08

**Authors:** Benjamin Schüz, Christopher Jones

**Affiliations:** 1 Institut für Public Health und Pflegeforschung, Universität Bremen, Grazer Straße 4, 28359 Bremen, Deutschland; 2 Leibniz-WissenschaftsCampus Digital Public Health, Bremen, Deutschland; 3 Zentrum für Präventivmedizin und Digitale Gesundheit (CPD), Medizinische Fakultät Mannheim der Universität Heidelberg, Mannheim, Deutschland

**Keywords:** Digitalisierung, Soziale Medien, Falschinformation, Desinformation, Gesundheitsinformationen, Digitalisation, Social media, Misinformation, Disinformation, Health information

## Abstract

Insbesondere im Kontext der COVID-19-Pandemie sind Falsch- und Desinformationen in sozialen Medien zu einer Herausforderung für effektive Public-health-Maßnahmen geworden. Hier betrachten wir Einflussfaktoren auf das Glauben und Teilen von Falschinformationen auf individueller, sozialer und situativ-kontextueller Ebene und diskutieren Interventionsmöglichkeiten.

Auf individueller Ebene spielen Wissensdefizite, mangelnde Kompetenzen und emotionale Motivation eine Rolle. Menschen mit geringer Gesundheitskompetenz und bestimmten Überzeugungen sind anfälliger für Falschinformationen. Auf sozialer Ebene beeinflussen die Glaubwürdigkeit von Informationsquellen und soziale Normen das Teilen von Falschinformationen. Das Bedürfnis nach Status und Gruppenzugehörigkeit spielt ebenfalls eine Rolle. Auf kontextueller Ebene wirken Emotionen und die Wiederholung von Nachrichten auf das Glauben und Teilen von Falschinformationen.

Interventionen auf individueller Ebene beinhalten Maßnahmen zur Verbesserung von Wissen und Kompetenzen. Auf sozialer Ebene können soziale Prozesse und soziale Normen angesprochen werden, um das Teilen von Falschinformationen zu reduzieren. Auf kontextueller Ebene wird die Regulierung von sozialen Netzwerken als wichtiger Ansatzpunkt betrachtet.

Es wird darauf hingewiesen, dass soziale Ungleichheiten in der Exposition gegenüber Falschinformationen und im Umgang damit eine wichtige Rolle spielen. Es bleibt unklar, ob die Anfälligkeit für Falschinformationen eine generelle Tendenz oder kontextabhängig ist. Die Entwicklung von Interventionen muss daher vielfältige Einflussfaktoren berücksichtigen.

## Abstract (en)

Misinformation and disinformation in social media have become a challenge for effective public health measures. Here, we examine factors that influence believing and sharing false information, both misinformation and disinformation, at individual, social, and contextual levels and discuss intervention possibilities.

At the individual level, knowledge deficits, lack of skills, and emotional motivation have been associated with believing in false information. Lower health literacy, a conspiracy mindset and certain beliefs increase susceptibility to false information. At the social level, the credibility of information sources and social norms influence the sharing of false information. At the contextual level, emotions and the repetition of messages affect belief in and sharing of false information.

Interventions at the individual level involve measures to improve knowledge and skills. At the social level, addressing social processes and social norms can reduce the sharing of false information. At the contextual level, regulatory approaches involving social networks is considered an important point of intervention.

Social inequalities play an important role in the exposure to and processing of misinformation. It remains unclear to which degree the susceptibility to belief in and share misinformation is an individual characteristic and/or context dependent. Complex interventions are required that should take into account multiple influencing factors.

## Einleitung

Eigentlich müssten die meisten Leser:innen zum Zeitpunkt der Lektüre dieses Artikels bereits tot sein – der Ballermann-Sänger Michael Wendler hat schließlich seinen über 140.000 Followern auf dem sozialen Messenger-Dienst Telegram bereits im August 2021 prophezeit: „!!LETZTE WARNUNG!! DR. COLDWELL SICHER: IM SEPTEMBER SIND ALLE GEIMPFTEN TOT“^1^. Auch noch nach dem September 2021 hatte derselbe Sänger wenig Hoffnung für die vielen Menschen, die sich mehrfach gegen COVID-19 impfen ließen: „!!JEDER COVID-19 GEIMPFTE WIRD INNERHALB VON 24 MONATEN STERBEN!!“^2^. Zur Beruhigung: Zum Zeitpunkt der Einreichung dieses Artikels (mehr als 24 Monaten nach dem letzten Post) waren beide Autoren dieses Artikels sowohl mehrfach gegen COVID-19 geimpft als auch noch am Leben.

Spaß und Häme beiseite, was diese Posts allerdings exemplarisch zeigen, ist, dass gesundheitliche Falsch- und Desinformation in sozialen Medien eine sehr große Reichweite erzielen können. Europaweite Umfragen [1] haben gezeigt, dass mehr als ein Drittel der Nutzer:innen von Social Media davon berichten, häufig bis täglich mit Falschinformationen konfrontiert zu sein. Sowohl Falschinformation (möglicherweise unbeabsichtigt falsch wiedergegebene oder inkorrekte Information) als auch Desinformation (intentional weitergegebene wissentlich falsche Information) zu gesundheitlichen Themen in sozialen Medien können verheerende Folgen für die populationsbezogene Gesundheit haben, insbesondere wenn durch die fehlerhaften Informationen Vertrauen in empfohlene und evidenzbasierte Maßnahmen wie Impfungen unterminiert oder wie im Fall der COVID-19-Pandemie Verhaltensempfehlungen wie das Tragen von Masken nicht mehr befolgt werden [2].

In diesem Artikel werden wir daher das Ausmaß von gesundheitlichen Falschinformationen in digitalen sozialen Medien untersuchen und anhand einer systematischen Betrachtung von Einflussgrößen auf das Glauben und die Weiterleitung von Falschinformationen Perspektiven für Interventionsmaßnahmen aufzeigen, um diesem aktuellen und relevanten Public-health-Problem [3] zu begegnen.

## Verbreitung von Falsch- und Desinformation

Das „International Fact-Checking Network“ (IFCN) der US-amerikanischen Journalistenschule „Poynter Institute“ hat im Kontext der COVID-19-Pandemie über 17.000 unterschiedliche Falschinformationen in den sozialen Medien dokumentiert [4], die in unzähligen weiteren Einzelnachrichten verbreitet oder geteilt wurden. Problematisch ist, dass sich Falschinformationen typischerweise schneller verbreiten als korrekte Information [5] und dann innerhalb eng verbundener sozialer Netzwerke um besonders prominente Accounts vielfach geteilt werden [6]. So ergeben sich, ähnlich wie bei Krankheitserregern, Szenarien, bei denen eine Falschmeldung exponentielle Verbreitung erreichen kann – daher auch die Analogie einer „Infodemie“ [7].

Analysen der bekanntesten Social-Media-Plattformen wie Twitter, Facebook oder Instagram zeigen, dass alle das Potenzial haben, eine exponentielle Verbreitung zu fördern – einige jedoch weit mehr als andere. Auf Twitter (heute: „X“) wurden beispielsweise laut einer Studie aus 2018 Falschinformationen etwa zu 70 % wahrscheinlicher geteilt als korrekte Informationen, und korrekte Informationen brauchten bis zu 6‑mal länger, um 1500 Menschen zu erreichen [5]. Das Potenzial für eine Infodemie hängt also stark von den Plattformen [3], aber auch vom Verhalten der Nutzer:innen ab. Normalerweise machen Falsch- und Desinformation nur einen kleinen Anteil des Informationsangebots auf Social Media aus, allerdings konsumieren und verbreiten einige wenige und sehr spezifische Gruppen den Großteil der Falschinformation – hier wird nicht nur von „Supersharern“, sondern zunehmend auch von „Superconsumern“ gesprochen [8]. Solche Cluster von Nutzer:innen können sich zunehmend polarisieren und dadurch die Verbreitung von Falschinformationen beschleunigen und mögliche Korrekturen erschweren [9].

Für den Konsum von Falschinformationen (bzw. den Glauben daran) und das Teilen von Falschinformationen gibt es möglicherweise unterschiedliche Einflussfaktoren. Im Folgenden betrachten wir deshalb beide Prozesse getrennt voneinander und ordnen die Einflussgrößen in einem sozialökologischen Modell mit unterschiedlichen hierarchisch angeordneten sozialen und umweltbezogenen Ebenen an [10].

## Glauben an Falsch- und Desinformationen: Einflussfaktoren

**Figure 1:**
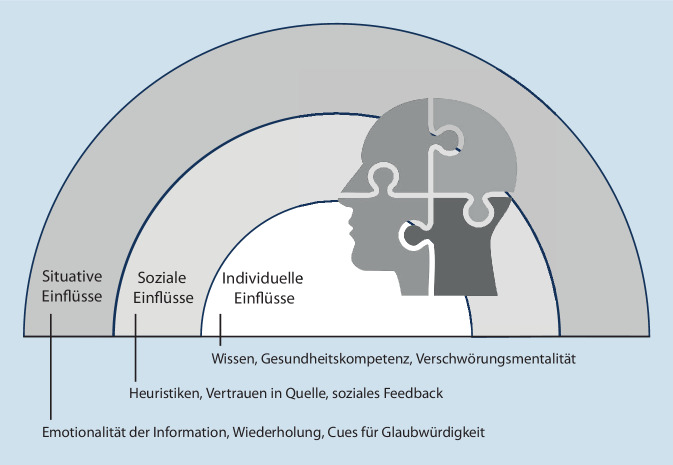

Insbesondere im Kontext der COVID-19-Pandemie wurde zunehmend an Einflussgrößen geforscht, die erklären können, warum und wie Personen Falsch- und Desinformationen in sozialen Medien Glauben schenken und welche Interventionsmöglichkeiten sich daraus ableiten lassen. Wir ordnen diese Einflussgrößen auf 3 Ebenen an (individuell, sozial und situativ-kontextbezogen; Abb. 1), was vor allem pragmatischen Überlegungen geschuldet ist – die Ebenen ließen sich sicherlich auch feiner unterteilen.

### Individuelle Einflussfaktoren.

Eine zentrale Annahme ist, dass Nutzer:innen Falsch- und Desinformationen schlicht aufgrund mangelnden Wissens, geringer Bildung, fehlender Kompetenz, zwischen vertrauenswürdigen und irreführenden Informationen zu unterscheiden, oder wegen erschwertem Zugang zu vertrauenswürdigen Informationen Glauben schenken [11]. So geht beispielsweise geringe Gesundheitskompetenz damit einher, dass Menschen Falschinformationen eher nicht als solche erkennen [12]. Ähnliche Befunde zeigen sich für andere Kompetenzbereiche, wie beispielsweise digitale (Gesundheits‑)Kompetenzen [13], Medienkompetenzen und naturwissenschaftliche Bildung [14]. Weil solche digitalen und Medienkompetenzen auch nach dem Alter ungleich verteilt sind, fällt es teilweise älteren Menschen schwerer, Falschinformationen zu identifizieren [15].

Neben individuellen Kompetenzen sind aber auch motivationale Faktoren relevant. Menschen glauben beispielsweise Falschinformationen eher, wenn diese mit bereits bestehenden Überzeugungen übereinstimmen [16]. Wenn solche Überzeugungen beinhalten, dass die Welt von mächtigen Personengruppen mit böswilligen Absichten im Verborgenen gelenkt wird, spricht man von Verschwörungsmentalität („conspiracy mindset“; [17]). Eine solche Überzeugung kann, insbesondere wenn der eigene Einfluss auf den Lauf der Dinge als gering eingeschätzt wird und die Informationen dem wissenschaftlichen Konsens widersprechen, dazu beitragen, dass zur Überzeugung passende Falschinformationen geglaubt werden. Nutzer:innen, die davon überzeugt sind, dass sie wichtige Ereignisse ohne eigene Suche mitbekommen (engl. „News-will-find-me-thinking“ [18]) oder stark intuitions- oder auch emotionsgeleitet über den Wahrheitsgehalt von Nachrichten entscheiden, sind ebenfalls eher anfällig für Fehlschlüsse [19]. Im Gegensatz dazu kann ein kritisch-reflektierender und prüfender kognitiver Stil (engl. „need for cognition“ [20]) dazu beitragen, vertrauenswürdige Informationen und den aktuellen wissenschaftlichen Konsens zu identifizieren [21, 22].

Allerdings kann auch eine gründlichere Beschäftigung (tiefere Verarbeitung – „deliberation“) mit Inhalten von Falschinformationen den Glauben an diese Inhalte verstärken. Dies gilt insbesondere für Verschwörungsinhalte, wenn diese mit bestehenden Einstellungen oder Ideologien der Nutzer:innen übereinstimmen. In einer Reihe von Experimenten konnten Bago et al. [23] zeigen, dass eine Intervention zur Förderung analytischen Denkens vor allem dazu führte, dass die Teilnehmer:innen neue Informationen in Einklang mit vorher bestehenden Einstellungen interpretierten: Teilnehmer:innen mit höherer Verschwörungsmentalität interpretierten neue Inhalte im Einklang mit bestehenden Verschwörungstheorien, Teilnehmer:innen mit niedriger Verschwörungsmentalität interpretierten neue Informationen im Widerspruch zu bestehenden Verschwörungstheorien.

### Soziale Einflussfaktoren.

Insbesondere wenn sich Menschen in informationsreichen Umgebungen wie sozialen Netzwerken bewegen, kommen Heuristiken (verkürzte Entscheidungsregeln) zum Einsatz [24]. Eine zentrale Rolle können dabei soziale Informationen wie die Glaubwürdigkeit der Informationsquelle spielen [25]. Wenig überraschend werden Informationen von Personen, die als glaubwürdig eingeschätzt werden, eher für richtig gehalten. Das gilt insbesondere auch für Nachrichten aus offiziellen Quellen, wo mehr Vertrauen in die Institutionen auch mehr Vertrauen in die Informationen bedingen kann [26]. Darüber hinaus erhöht soziales Feedback die Vertrauenswürdigkeit von Informationen – mehr Likes können also auch offensichtliche Falschinformationen vertrauenswürdiger machen [27].

### Situative und kontextuelle Einflussfaktoren.

Weiter können situativ variable Faktoren innerhalb der Person und in den kontextuellen Rahmenbedingungen von Nachrichten entscheidend dafür sein, ob die Falschinformationen geglaubt werden. Insbesondere haben die durch eine Nachricht angesprochenen Emotionen einen Einfluss darauf, ob Falschinformationen erkannt werden [28]. Der momentane Affekt der Nutzer:innen kann sich auch auf den Glauben an Falschinformationen auswirken: Wer bei einer Entscheidungsaufgabe stärkeren Affekt empfindet (negativ oder positiv), glaubt eher falsche Informationen. Und die Anweisung, sich bei der Einschätzung auf die eigenen Emotionen zu verlassen, erhöhte in einer Studie noch zusätzlich den Glauben an falsche Überschriften [29].

Daneben haben auch situative Merkmale aus dem Kontext der Falschinformationen wie die Wiederholung von Nachrichten einen Einfluss. Wird beispielsweise eine Überschrift wiederholt angezeigt, neigen Nutzer:innen eher dazu, diese auch zu glauben (engl. „illusory truth effect“ [30]), solange sich die Information auch nur entfernt plausibel anhört [31].

## Teilen von Falsch- und Desinformationen: Einflussfaktoren

**Figure 2:**
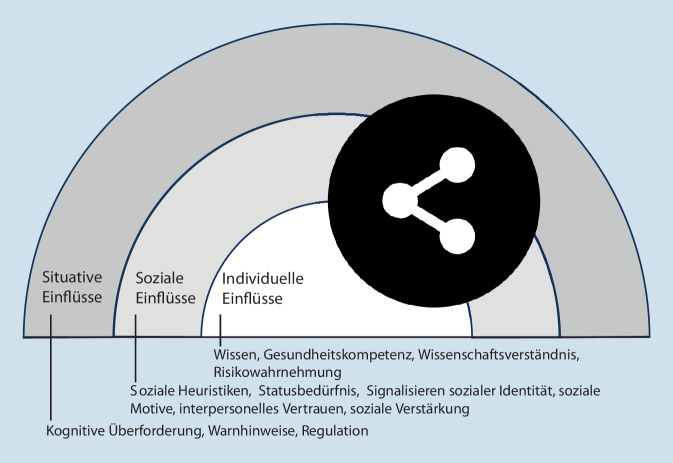

Auch wenn es plausibel scheint, dass Falschinformationen, als solche erkannt, weniger wahrscheinlich weitergeleitet werden, zeigen aktuelle Arbeiten, dass es für das Teilen oder Weiterleiten eines Posts weniger entscheidend ist, ob der Inhalt als korrekt oder falsch erkannt wird, als anzunehmen wäre [14]: In dieser Studie von Pennycook und Kollegen schätzten die Teilnehmer:innen Überschriften, die dem wissenschaftlichen Konsens entsprachen, tatsächlich auch häufiger als richtig ein als frei erfundene Überschriften. Allerdings hatte diese Einschätzung fast gar keinen Einfluss auf ihre Absicht, die Nachrichten zu teilen, unabhängig davon, ob es gesundheitliche oder politische Informationen waren [14]. Dieses Missverhältnis zwischen der inhaltlichen Bewertung und der Absicht zu teilen deutet darauf hin, dass viele Teilnehmer:innen anscheinend bereit waren, Inhalte zu teilen, die sie selbst nicht als vollständig zutreffend beurteilt hatten. Daher betrachten wir im Folgenden die Faktoren, die das Teilen beeinflussen und ordnen diese ebenfalls den Ebenen innerhalb eines sozialökologischen Modells zu (Abb. 2).

### Individuelle Einflussfaktoren.

Wissen – oder vor allem fehlendes Wissen – hat einen Einfluss auf das Weiterleiten von Falschinformationen. Personen mit geringerem gesundheitlichen Vorwissen teilten Falschinformationen eher als Personen mit mehr Vorwissen [32]. Ein besseres Verständnis von wissenschaftlichen Abläufen führte eher dazu, dass weniger Falschinformationen geteilt wurden [33]. Auch bessere Gesundheits- und digitale Kompetenzen sind damit assoziiert, dass Menschen weniger Falschinformationen teilen [34]. Neben diesem Wissen bedingen auch Faktoren aus Theorien des Gesundheitsverhaltens wie Risikowahrnehmung das Teilen von Falschinformationen – allerdings in Abhängigkeit von Fachwissen: Während gut informierte Teilnehmer:innen mit höherer Risikowahrnehmung eher weniger Falschinformationen teilten, teilten schlechter informierte eher mehr Falschinformationen, wenn sie gesundheitliche Risiken wahrnahmen [35].

### Soziale Einflussfaktoren.

Das Teilen von Information in sozialen Medien hat allerdings auch zentrale soziale Funktionen. So kann das Bedürfnis, Status [36], Gruppenzugehörigkeit [37] oder soziale Identität [38] zu signalisieren, dazu führen, dass Falschinformationen geteilt werden. Dabei scheint die Übereinstimmung mit persönlichen Werten und Ansichten viel stärkeren Einfluss auf das Teilen als auf das Glauben von Falschinformationen auszuüben. So zeigen politisch eher konservativ orientierte Nutzende eine insgesamt geringere Tendenz, Informationen zu teilen, sie neigen jedoch dazu, viel eher irreführende als zutreffende Informationen zu teilen [14]. Soziale Hinweisreize (z. B. Likes aus dem eigenen sozialen Netzwerk) können das Verhalten, Inhalte zu teilen, zusätzlich verstärken. Analog können aber soziale Informationen, dass im eigenen Netzwerk bestimmte Informationen nicht geteilt oder „gelikt“ werden, auch dazu führen, dass weniger Falschinformationen geteilt werden [39]. Darüber hinaus können eigentlich positive soziale Motive wie der Wunsch, andere in seinem sozialen Netzwerk auf mögliche Risiken hinzuweisen, dazu führen, dass mehr Falschinformationen verbreitet werden. Zusammenfassend lässt sich feststellen, dass soziale Einflussgrößen vermutlich einen größeren Einfluss auf das Teilen von Falschinformationen haben als individuelle Einflussgrößen und sich daher auch eher als Interventionsziele anbieten.

### Situative und kontextuelle Einflussfaktoren.

Allerdings lässt sich auch unter Einbezug von individuellen und sozialen Einflussgrößen das Teilen von Falsch- und Desinformationen nicht vollständig erklären [21]. Deswegen kann es hilfreich sein, auch hier situative und kontextbezogene Merkmale der Social-Media-Plattformen zu betrachten.

Zum Geschäftsmodell der Plattformen gehört die Maximierung der Zeit, die Nutzer:innen auf der Plattform verbringen, und entsprechend die Maximierung ihrer dortigen Aktivitäten (Likes, Reposts oder allgemeines „user engagement“), um so mehr gezielte Werbung zeigen zu können. Die informationsreiche Umgebung kann, wie oben erwähnt, dazu führen, dass Nutzer:innen ihr Verhalten auf den Plattformen hauptsächlich auf Heuristiken aufbauen und sich weniger intensiv mit den eigentlichen Inhalten von Posts beschäftigen.

Dies zeigt sich beispielsweise, wenn Studienteilnehmer:innen, die sich von der hohen Informationsmenge (engl. „cognitive overload“) überfordert fühlen, auch eher bereit sind Falschinformationen zu teilen [40]. In Experimenten konnte gezeigt werden, dass bereits kurz eingeblendete Nachfragen, ob die Nutzer:innen die Information als richtig einschätzen, dazu führen konnten, dass sich die Nutzer:innen intensiver mit der Nachricht auseinandersetzen. Der veränderte Aufmerksamkeitsfokus führte dazu, dass die Informationen seltener geteilt wurden [14].

## Interventionsansätze: Besseres Erkennen von Falschinformationen und weniger Teilen

Analog zu der Anordnung der Einflussgrößen auf hierarchischen Ebenen können auch Interventionen auf individuelle, soziale und kontextuelle Ansatzpunkte abzielen.

### Individuelle Ebene.

Interventionen auf individueller Ebene können das Wissen und die Kompetenzen von Nutzer:innen in den sozialen Medien verbessern, um so die Identifikation von Falsch- und Desinformation zu erleichtern. Solche Interventionen knüpfen an die oben erwähnte Defizithypothese an und sollen dazu führen, dass Nutzer:innen die notwendigen Kompetenzen erwerben, wahre von falschen Informationen zu trennen und so weniger Falschinformationen weiterzuleiten. Dieser kompetenzorientierte Ansatz entstand vor allem aus der Erkenntnis, dass eine Richtigstellung von Falschinformationen im Nachhinein (sog. Debunking) mit einer Reihe von Schwierigkeiten verbunden und nur in sehr begrenztem Rahmen effektiv ist [41].

Präventive Ansätze zur Bekämpfung von Falschinformationen werden häufig als Prebunking (vorgreifendes Debunking) bezeichnet. Das prominenteste Beispiel solchen Prebunkings sind Interventionen, die sich einer Impfanalogie bedienen (engl. Inoculation). Dabei sollen die Nutzenden durch die Exposition gegenüber eher „schwachen“ Falschinformationen auf die spätere Konfrontation mit tatsächlich irreführenden Inhalten vorbereitet werden [42]. Wichtig dabei ist, dass die Nutzenden den irreführenden Beiträgen nicht einfach ausgesetzt werden, sondern diesen jeweils ein Hinweis auf nun folgende Falschinformationen und eine differenzierte Gegendarstellung vorangehen. Neben dieser eher *passiven* Art der inhaltlichen Auseinandersetzung [43] unterstützen *aktive* Immunisierungsinterventionen die Nutzenden dabei, eigene Gegenargumente auszuformulieren [44].

Während bei der Entwicklung der Immunisierungsinterventionen zunächst vor allem auf sehr spezifische und konkrete Fälle von Falschinformationen eingegangen wurde (z. B. auf die Falschinformation, dass das Medikament Ivermectin bei der Behandlung von COVID-19 große Erfolge erzielt habe), werden aktuell eher Interventionen entwickelt, die typische Charakteristika der Formulierung, Darstellung und Argumentation von Falschinformationen beschreiben. So sollen die Nutzenden befähigt werden, typische Muster zu erkennen, die dann auf irreführende Inhalte zu verschiedenen Themen übertragen werden können. Diese Art der strategieorientierten Immunisierungsintervention kann vor allem dann hilfreich sein, wenn neue und noch unbekannte Inhalte auftauchen. Sind die angesprochenen Themen und genutzten Argumentationsstränge der Falschinformationen bereits bekannt, können eher inhaltlich orientierte Interventionen stärkere Effekte vorweisen. Zwar haben Metaanalysen [45] zuletzt positive Effekte von Immunisierungsinterventionen bestätigt, allerdings bleibt bisher unklar, ob diese Effekte aus einer grundlegend skeptischen Haltung gegenüber allen Informationen entstehen oder spezifisch für irreführende Inhalte zu interpretieren sind.

### Soziale Ebene.

Interventionen auf sozialer Ebene zielen vor allem darauf ab, durch die Veränderung von sozialen Prozessen das Teilen von Falschinformationen zu beeinflussen. So konnte in einer Interventionsstudie [39] gezeigt werden, dass durch Veränderungen in den sozialen Rückmeldungen Veränderungen im Teilen verursacht wurden. Statt Likes und Shares wurde angezeigt, wie viele Mitglieder des eigenen Netzwerks (Followers) oder des gesamten sozialen Netzwerks eine Falschinformation zwar gesehen, aber nicht gelikt oder weitergeleitet hatten. Wenn Falschinformationen mit der Information versehen wurden, dass sie nur von wenigen Mitgliedern des eigenen Netzwerks geteilt wurden, sank die Wahrscheinlichkeit des Teilens deutlich. Dieser Effekt ist möglicherweise durch Veränderungen in sozialen Normen bedingt, d. h., die Nutzer:innen nahmen es als weniger angebracht wahr, solche Inhalte zu teilen.

### Situative und kontextuelle Ebene: Regulation sozialer Netzwerke.

Auch wenn in Deutschland seit 2018 das Netzwerkdurchsetzungsgesetz die Betreiber von sozialen Medien verpflichtet, vor allem Anfeindungen und Beleidigungen (Hate Speech) innerhalb von 24 h zu entfernen, ist die Durchführung kontrovers diskutiert worden. Auf Ebene der Europäischen Union (EU) gilt seit 2022 der Digital Services Act, der zum einen vorsieht, dass Plattformbetreiber regelmäßig Bericht darüber erstatten, inwiefern Maßnahmen zur Vermeidung von illegalen Inhalten und Falschinformationen durchgeführt wurden,^3^ zum anderen aber auch erlaubt, dass im Kontext von Krisen das „European Board for Digital Services“ von Betreibern verlangen kann, bestimmte Inhalte zu filtern und inhaltliche Moderation durchzuführen [46].

Im Rahmen der aktuellen Berichterstattung fällt vor allem auf, dass seit der Übernahme von Twitter (jetzt „X“) durch Elon Musk und der damit einhergehenden Entsperrung von vielen Accounts, die zuvor durch Desinformation und Hate Speech aufgefallen waren, wieder deutlich mehr Desinformation veröffentlicht wird. Auch der anfangs erwähnte Ballermann-Sänger durfte seinen Account wieder eröffnen. Vor allem die Veränderungen auf dieser Plattform zeigen, wie wichtig ernst gemeinte Regulation und inhaltliche Moderation zur Vermeidung von Falsch- und Desinformation im Netz sind.

## Offene Fragen zu sozialer Ungleichheit und individuellen Unterschieden

### Soziale Ungleichheit.

Ein besonders relevanter, aber oft nicht mitberücksichtigter Aspekt in der Forschung zu gesundheitsbezogenen Falschinformationen im Netz und deren Implikationen sind soziale Ungleichheiten in der Exposition und in der Verteilung von Ressourcen zur Unterscheidung zwischen falschen und korrekten Informationen [47]. Soziale Unterschiede in Grundlagen- und Anwendungswissen zu Gesundheitsthemen haben erwiesenermaßen einen Einfluss darauf, wie Menschen mit Gesundheitsinformationen umgehen – die „Knowledge Gap Hypothesis“ [48] sagt beispielsweise aus, dass Menschen mit höheren Bildungsabschlüssen und mehr gesundheitsbezogenem Grundlagenwissen weniger Probleme damit haben, wahre von falschen Informationen zu unterscheiden (was insbesondere in globalen Notfalllagen wie einer Pandemie wichtig ist; [49]), und zudem schlüssige Empfehlungen eher als Menschen mit niedrigen Bildungsabschlüssen befolgen. Gleichzeitig zeigt eine Reihe von Untersuchungen während der COVID-19-Pandemie, dass Menschen mit geringeren Bildungsabschlüssen sich mehr oder sogar exklusiv auf soziale Medien als Quelle für Nachrichten und Gesundheitsinformationen verlassen und damit auch anfälliger dafür sind, Falsch- und Desinformation ausgesetzt zu werden.

Während der Pandemie haben sich auch Muster gezeigt, wonach Menschen in sozialen Medien Informationen vor allem von solchen Quellen und Persönlichkeiten bezogen und weiterleiteten, die ihnen im Hinblick auf demografische Merkmale und politische Ausrichtung ähnlich waren [50]. So zeigten sich beispielsweise in Großbritannien Unterschiede in der Impfbereitschaft bei Frauen aus ethnischen Minderheiten im Vergleich zur Allgemeinbevölkerung [51]. Grund dafür war, dass diese Frauen Gesundheitsinformationen aus Kanälen sozialer Netzwerke bezogen, in denen mehr Falschinformationen über mögliche Auswirkungen der COVID-19-Schutzimpfung auf die Fruchtbarkeit zirkulierten. Aus dieser Studie war aber auch ersichtlich, dass Frauen, die Quellen von Gesundheitsinformationen außerhalb sozialer Netzwerke vertrauten, eine deutlich höhere Impfbereitschaft zeigten. Gleichzeitig ist es wichtig zu betonen, dass neben der unterschiedlichen Exposition gegenüber Falschinformationen auch Diskriminierungserfahrungen im Gesundheitssystem und ein damit verbundenes geringeres Vertrauen in Informationen der entsprechenden Institutionen eine Rolle spielen können [52].

Fact-checking, also die Überprüfung, ob Informationen in sozialen Netzwerken korrekt sind, läuft oft über KI-basierte Systeme ab. Erste Simulationsstudien zeigen, dass die Resultate von Fact-checking Nutzer:innen aus benachteiligten Bevölkerungsgruppen weniger zugutekommen, insbesondere wenn Falschinformationen in seltener benutzten Sprachen oder in anderen Formaten erst später oder gar nicht überprüft werden [53].

### Individuelle Unterschiede.

Bisher ist unklar, ob die Anfälligkeit für Falschinformationen eine generelle individuelle Tendenz darstellt oder kontext- und themenabhängig auftritt. Es ist anzunehmen, dass bei Nutzer:innen, die politische Falschinformationen glauben, eine generelle individuelle Tendenz besteht, gleichzeitig auch gesundheitsbezogene Falschinformationen zu glauben und zu teilen. Dies wird beispielsweise durch Befunde gestützt, die bei Personen mit einer Verschwörungsmentalität eine größere generelle Bereitschaft zum Teilen von Falschinformationen zeigen [17]. Ob und welche als falsch erkannte Informationen geteilt werden, kann aber auch, wie oben erwähnt, davon abhängen, inwiefern diese Informationen mit eigenen Überzeugungen übereinstimmen. [16]. Ein besseres Verständnis dieser Zusammenhänge könnte die Entwicklung und Erprobung von Interventionen verbessern und effizienter machen. So könnten generelle Kompetenzen zentrales Interventionsziel sein und gleichzeitig in Abhängigkeit von individuellen Merkmalen zusätzlich soziale und kontextuelle Ansatzpunkte adressiert werden.

## Schlussfolgerungen

Falsch- und Desinformation in sozialen Medien sind ein relevantes Problem. Insbesondere im Kontext der COVID-19-Pandemie hat das Thema zunehmend wissenschaftliche und mediale Aufmerksamkeit bekommen. Im Zuge dieser zunehmenden Aufmerksamkeit wurde eine Reihe von Interventionsansätzen vorgestellt, die an verschiedenen individuellen, sozialen und kontextuellen Punkten ansetzen [22].

Es handelt sich aber um ein komplexes Problem mit Einflussgrößen auf Ebene der Person, des sozialen Umfelds, der Situation und des regulativen Kontexts. In welchem Maße Menschen aufgrund von individuellen Unterschieden, Lebenslagen und Ressourcen oder wegen situativer und kontextabhängiger Faktoren für Falschinformationen anfällig sind, ist noch nicht abschließend geklärt. Die teilweise noch sehr disparate Literatur dazu, wie wir Falschinformationen und Desinformationen in sozialen Medien verarbeiten, interpretieren und womöglich auch weiterleiten, lässt sich vermutlich noch nicht in einem integrierten Modell von maladaptivem Engagement mit Falschinformationen zusammenfassen. Es gibt aber sicherlich kein Allheilmittel gegen Falsch- und Desinformation in digitalen sozialen Medien und wir werden komplexe Interventionen entwickeln müssen, die verschiedene Einflussgrößen kombinieren.

## Footnotes

1 https://twitter.com/AttilaIncognito/status/1607420777011478528, letzter Zugriff 12.01.2024.

2 https://web.archive.org/web/20210602163906/https://t.me/s/MICHAELWENDLEROFFICIAL, letzter Zugriff 12.01.2024.

3 Der aktuelle Bericht aus dem Jahr 2023 kann beispielsweise hier abgerufen werden: https://disinfocode.eu/reports-archive/?years=2023.

## References

[1] European Union Fake news and disinformation online—März 2018—Eurobarometer survey. https://europa.eu/eurobarometer/surveys/detail/2183. Zugegriffen: 29. Sept. 2023

[2] Imhoff R, Lamberty P (2020). A bioweapon or a hoax? The link between distinct conspiracy beliefs about the coronavirus disease (COVID-19) outbreak and pandemic behavior. Soc Psychol Personal Sci.

[3] Cinelli M, Quattrociocchi W, Galeazzi A (2020). The COVID-19 social media infodemic. Sci Rep.

[4] CoronaVirusFacts Alliance. Poynter. https://www.poynter.org/coronavirusfactsalliance/. Zugegriffen: 26. Jan. 2024

[5] Vosoughi S, Roy D, Aral S (2018). The spread of true and false news online. Science.

[6] Shao C, Hui P-M, Wang L (2018). Anatomy of an online misinformation network. PLoS One.

[7] Zarocostas J (2020). How to fight an infodemic. Lancet.

[8] Guess AM, Malhotra N, Pan J (2023). How do social media feed algorithms affect attitudes and behavior in an election campaign?. Science.

[9] Zollo F, Bessi A, Vicario MD (2017). Debunking in a world of tribes. PLoS ONE.

[10] Sundelson AE, Jamison AM, Huhn N, Pasquino S-L, Sell TK (2023). Fighting the infodemic: the 4 i framework for advancing communication and trust. BMC Public Health.

[11] Scherer LD, Pennycook G (2020). Who is susceptible to online health misinformation?. Am J Public Health.

[12] Scherer LD, McPhetres J, Pennycook G (2021). Who is susceptible to online health misinformation? A test of four psychosocial hypotheses. Health Psychol.

[13] Pickles K, Cvejic E, Nickel B (2021). COVID-19 misinformation trends in Australia: prospective longitudinal national survey. J Med Internet Res.

[14] Pennycook G, McPhetres J, Zhang Y, Lu JG, Rand DG (2020). Fighting COVID-19 misinformation on social media: experimental evidence for a scalable accuracy-nudge intervention. Psychol Sci.

[15] Brashier NM, Schacter DL (2020). Aging in an era of fake news. Curr Dir Psychol Sci.

[16] Sikder O, Smith RE, Vivo P, Livan G (2020). A minimalistic model of bias, polarization and misinformation in social networks. Sci Rep.

[17] Romer D, Jamieson KH (2023) The role of conspiracy mindset in reducing support for child vaccination for COVID-19 in the United States. Front Psychol 14:10.3389/fpsyg.2023.1175571 PMC10294680 37384178

[18] Lin Y, Chen M, Lee SY (2024). Understanding the effects of news-finds-me perception on health knowledge and information seeking during public health crises. Health Commun.

[19] Roozenbeek J, Schneider CR, Dryhurst S (2020). Susceptibility to misinformation about COVID-19 around the world. R Soc open sci.

[20] Ozimek P, Nettersheim M, Rohmann E, Bierhoff H-W (2022). Science vs. conspiracy theory about COVID-19: need for cognition and openness to experience increased belief in conspiracy-theoretical postings on social media. Behav Sci.

[21] Pennycook G, Rand DG (2021). The psychology of fake news. Trends Cogn Sci.

[22] Roozenbeek J, Culloty E, Suiter J (2023). Countering misinformation. Eur Psychol.

[23] Bago B, Rand DG, Pennycook G (2022). Does deliberation decrease belief in conspiracies?. J Exp Soc Psychol.

[24] Pennycook G, Rand DG (2019). Lazy, not biased: susceptibility to partisan fake news is better explained by lack of reasoning than by motivated reasoning. Cognition.

[25] Swire-Thompson B, Lazer D (2020). Public health and online misinformation: challenges and recommendations. Annu Rev Public Health.

[26] Nadarevic L, Reber R, Helmecke AJ, Köse D (2020). Perceived truth of statements and simulated social media postings: an experimental investigation of source credibility, repeated exposure, and presentation format. Cogn Res Princ Implic.

[27] Butler L, Fay N, Ecker U (2022). Social endorsement influences the continued belief in corrected misinformation.

[28] Freiling I, Krause NM, Scheufele DA, Brossard D (2023). Believing and sharing misinformation, fact-checks, and accurate information on social media: the role of anxiety during COVID-19. New Media Soc.

[29] Martel C, Pennycook G, Rand DG (2020). Reliance on emotion promotes belief in fake news. Cogn Res Princ Implic.

[30] Pennycook G, Rand DG (2020). Who falls for fake news? The roles of bullshit receptivity, overclaiming, familiarity, and analytic thinking. J Pers.

[31] Pennycook G, Cannon TD, Rand DG (2018). Prior exposure increases perceived accuracy of fake news. J Exp Psychol Gen.

[32] Xue H, Taylor L (2023). When do people believe, check, and share health rumors on social media? Effects of evidence type, health literacy, and health knowledge. J Health Psychol.

[33] Keselman A, Arnott Smith C, Leroy G, Kaufman DR (2021). Factors influencing willingness to share health misinformation videos on the internet: web-based survey. J Med Internet Res.

[34] Southwell BG, Otero Machuca J, Cherry ST, Burnside M, Barrett NJ (2023). Health misinformation exposure and health disparities: observations and opportunities. Annu Rev Public Health.

[35] Wang R, Zhang H (2023). Who spread COVID-19 (mis)information online? Differential informedness, psychological mechanisms, and intervention strategies. Comput Human Behav.

[36] Apuke OD, Omar B (2020). Modelling the antecedent factors that affect online fake news sharing on COVID-19: the moderating role of fake news knowledge. Health Educ Res.

[37] Lobato EJC, Powell M, Padilla LMK, Holbrook C (2020). Factors predicting willingness to share COVID-19 misinformation. Front Psychol.

[38] Shin J, Yang A, Liu W, Kim MH, Zhou A, Sun J (2022). Mask-wearing as a partisan issue: social identity and communication of party norms on social media among political elites. Soc Media Soc.

[39] Jones CM, Diethei D, Schöning J, Shrestha R, Jahnel T, Schüz B (2023). Impact of social reference cues on misinformation sharing on social media: series of experimental studies. J Med Internet Res.

[40] Wu M (2022). What drives people to share misinformation on social media during the COVID-19 pandemic: a stimulus-organism-response perspective. Int J Environ Res Public Health.

[41] van der Linden S, Roozenbeek J, Maertens R (2021). How can psychological science help counter the spread of fake news?. Span J Psychol.

[42] van der Linden S (2022). Misinformation: susceptibility, spread, and interventions to immunize the public. Nat Med.

[43] Basol M, Roozenbeek J, Berriche M, Uenal F, McClanahan WP, van der Linden S (2021). Towards psychological herd immunity: cross-cultural evidence for two prebunking interventions against COVID-19 misinformation. Big Data Soc.

[44] Traberg CS, Roozenbeek J, van der Linden S (2022). Psychological inoculation against misinformation: current evidence and future directions. Ann Am Acad Pol Soc Sci.

[45] Lu C, Hu B, Li Q, Bi C, Ju X-D (2023). Psychological Inoculation for credibility assessment, sharing intention, and discernment of misinformation: systematic review and meta-analysis. J Med Internet Res.

[46] Europäisches Parlament VERORDNUNG (EU) 2022/2065 Des Europäischen Parlaments und des Rates vom 19. Oktober 2022 über einen Binnenmarkt für digitale Dienste und zur Änderung der Richtlinie 2000/31/EG. Gesetz über digitale Dienste

[47] Viswanath K, Lee EWJ, Pinnamaneni R (2020). We need the lens of equity in COVID-19 communication. Health Commun.

[48] Viswanath K, Finnegan JR (1996). The knowledge gap hypothesis: twenty-five years later. Ann Int Commun Assoc.

[49] Lin L, Savoia E, Agboola F, Viswanath K (2014). What have we learned about communication inequalities during the H1N1 pandemic: a systematic review of the literature. BMC Public Health.

[50] Gallagher RJ, Doroshenko L, Shugars S, Lazer D, Foucault Welles B (2021). Sustained online amplification of COVID-19 elites in the United States. Soc Media Soc.

[51] Naqvi M, Li L, Woodrow M, Yadav P, Kostkova P (2022) Understanding COVID-19 vaccine hesitancy in ethnic minorities groups in the UK. Front Public Health 10:10.3389/fpubh.2022.917242 PMC9284000 35844884

[52] Khan MS, Ali SAM, Adelaine A, Karan A (2021). Rethinking vaccine hesitancy among minority groups. Lancet.

[53] Neumann T, Wolczynski N (2023). Does AI-Assisted Fact-Checking Disproportionately Benefit Majority Groups Online?. Proc. 2023 ACM Conf. Fairness Account. Transpar.

